# Treatment Recommendations for Chronic Myeloid Leukemia

**DOI:** 10.4084/MJHID.2014.005

**Published:** 2014-01-02

**Authors:** Michele Baccarani, Fausto Castagnetti, Gabriele Gugliotta, Francesca Palandri, Gianantonio Rosti

**Affiliations:** 1 Department of Hematology and Oncology “L. and A. Seràgnoli”, University of Bologna, Bologna, Italy; 2 Institute of Hematology “L. and A. Seràgnoli”, Department of Specialty, Diagnostic and Experimental Medicine, University of Bologna, S.Orsola-Malpighi University Hospital, Bologna, Italy.

## Abstract

The first treatment of chronic myeloid leukemia (CML) included spleen x-radiation and conventional drugs, mainly Busulfan and Hydroxyurea. This therapy improved the quality of life during the chronic phase of the disease, without preventing nor significantly delaying the progression towards advanced phases. The introduction of allogeneic stem cell transplantation (alloSCT) marked the first important breakthrough in the evolution of CML treatment, because about 50% of the eligible patients were cured. The second breakthrough was the introduction of human recombinant interferon-alfa, able to achieve a complete cytogenetic remission in 15% to 30% of patients, with a significant survival advantage over conventional chemotherapy. At the end of the last century, about 15 years ago, all these treatments were quickly replaced by a class of small molecules targeting the tyrosine kinases (TK), which were able to induce a major molecular remission in most of the patients, without remarkable side effects, and a very prolonged life-span. The first approved TK inhibitor (TKI) was Imatinib Mesylate (Glivec or Gleevec, Novartis). Rapidly, other TKIs were developed tested and commercialized, namely Dasatinib (Sprycel, Bristol-Myers Squibb), Nilotinib (Tasigna, Novartis), Bosutinib (Busulif, Pfizer) and Ponatinib (Iclusig, Ariad). Not all these compounds are available worldwide; some of them are approved only for second line treatment, and the high prices are a problem that can limit their use. A frequent update of treatment recommendations is necessary. The current treatment goals include not only the prevention of the transformation to the advanced phases and the prolongation of survival, but also a length of survival and of a quality of life comparable to that of non-leukemic individuals. In some patient the next ambitious step is to move towards a treatment-free remission. The CML therapy, the role of alloSCT and the promising experimental strategies are reviewed in the context of the new therapeutic goals.

## Introduction

The first effective treatment of chronic myeloid leukemia (CML) included x-radiation to the spleen and conventional chemotherapeutic drugs, mainly Busulfan (BUS) and Hydroxyurea (HU). This therapy helped to limit the expansion of the myeloid tissue, improved significantly the quality of life during the chronic phase (CP) of the disease, but did neither prevent nor delay significantly the progression towards accelerated and blastic phase (AP, BP), with a limited effect on overall survival (OS).^1^–^4^ The introduction of allogeneic stem cell transplantation (alloSCT) marked the first important breakthrough in the evolution and the outcome of CML, because about 50% of the patients who were eligible for alloSCT became Philadelphia–negative, BCR-ABL negative, and were cured. Unfortunately, the best success of allo SCT were in patients less than 40 years old, while the median age at diagnosis is close to 60 years, and the cure was frequently linked to development of a chronic graft-versus-host-disease (cGVHD).^1^,^2^,^5^–^7^

The second breakthrough in therapy was the introduction of human recombinant interferon-alfa (rIFNα), that was able to achieve a complete cytogenetic remission in 15% to 30% of patients, and provided a significant survival advantage over conventional chemotherapy.^1^,^2^,^8^ At the end of the last century, about 15 years ago, all these treatments were quickly displaced by the discovery of a class of small molecules targeting the tyrosine kinases (TK), particularly the BCR-ABL TK, that is the cause of the leukemic transformation and the leukemic characteristics of Ph+ hematopoietic stem cells.^2^ The first approved TK inhibitor (TKI) was Imatinib Mesylate (Glivec or Gleevec, Novartis), that is still the CML standard treatment for many patients.^1^,^6^,^9^,^10^ Rapidly, other TKIs were developed tested and commercialized, namely Dasatinib (Sprycel, Bristol-Myers Squibb), Nilotinib (Tasigna, Novartis), Bosutinib (Busulif, Pfizer) and Ponatinib (Iclusig, Ariad).^11^–^17^ Not all these compounds are available worldwide; some of them are approved only for second line treatment, and the high prices are a problem that can limit their use.^18^ However, overall they provide an extraordinary inventory of active agents which we should learn to use to optimize the treatment of CML, with the purpose of avoiding death from leukemia, but also avoiding deaths and complications from treatment, improving the quality of life, achieving a cure, and also making better and proper use of the financial resources, that are never unlimited.

## The Goals of Treatment

Until the introduction of the TKIs, the goals of CML treatment were the prolongation of survival and, only for the patients eligible to alloSCT, the cure. Nowadays, the goals are more complex and more ambitious.^6^,^19^,^20^ If they are listed in a logical order, number one is the prevention of the progression towards accelerated phase (AP) and blastic phase (BP), to reduce to zero the risk of death for leukemia. Number two is to attain a length of survival comparable to that of non-leukemic people and a quality of life as close as possible to that of non-leukemic individuals. To achieve this latter objective, look not sufficient to clear the risk of dying of leukemia, but it is also necessary to avoid all deaths and complications related to the treatment, by limiting as much as possible the side-effects of the treatment while ensuring the compliance of the patients. At the same time, the patients should be assured to be no longer afraid of leukemia. In order to achieve all these goals, it is necessary to establish a specific professional network, because the disease is rare (1 to 1.5 new cases per year per 100.000 people) and a generalist can see only few such patients during his/her professional life. The management of CML should be modeled in a similar way to the management of diabetes mellitus type 1, where patient care is based on the cooperation between a specialized centre and the home physicians.

## The Time of Treatment

CML is a chronic disease. About 50% of patients are more than 60 years old, and about 50% of patients are asymptomatic. About 40% of patients are at a low risk of dying during the first two or three years, in absence of any affective treatment.^2^–^4^,^6^ Does that mean that the treatment can be delayed, until the disease becomes symptomatic? That’s the current policy in several chronic diseases like chronic lymphocytic leukemia and multiple myeloma, where there is not yet full evidence that the balance between early treatment and result is positive. But this is not the case in CML. In CML, treatment, whatever it may be, must be positioned as early as possible, to stop the process leading to progression to AP and BP. With minor and few exceptions, the battle can be victorious only if it is fought as early as possible.

## Drugs and Stem Cell Transplantation

Most all the cytotoxic anticancer agents have some degree of efficacy in CML. Radiations was abandoned long time ago. BUS should no longer be used, with very few and temporary exceptions. HU, that has a rapid effect, a low cost, and a favorable safety profile, is still used to control the disease in the first few days waiting for the results of diagnostic tests, and occasionally and temporarily in patients who cannot take TKIs or are resistant to all TKIs. Anagrelide is not a drug for CML, but it is used sometimes to limit a very high platelet count.^21^ rIFNα was the standard of care, competing with allo SCT, for about 10 years, until the introduction of imatinib.^1^,^2^ Although the results of rIFNα were considerable, particularly in low risk patients, this agent has been almost completely replaced by TKIs in the first line treatment, and is not recommended second-line, in the patients who fail the TKIs.^6^,^21^ However, the combination of rIFNα with TKIs hold strong promises and is currently tested in prospective studies.^22^–^24^ AlloSCT was the first line treatment in the eligible patients for many years, now more than thirty, and is still the only treatment that can ensure a true cure, that is to say a complete and stable molecular negativity.^7^ Still, alloSCT is the best available treatment after progression to AP and BP. The argument of alloSCT in CML is not covered in this issue, and the reader is referred to several recent reports and reviews.^1^,^5^–^7^,^25^–^27^ To summarize, although nowadays the upper age of transplant is up to 70 years, and in most cases it is possible to find a suitable donor also outside the family, although the risk of transplant-related mortality is substantially reduced, although the use of non-myeloablative regimens has helped to reduce transplant-related mortality and morbidity particularly in the elderly, the overall risk of mortality andcGVHD, in a population with a median age close to 60, are still hard to accept. Therefore, the current indication for allo SCT is TKIs-resistance in CP, and progression to AP and BP.^6^,^21^ Unfortunately, the success of allo SCT is limited in AP and is very small in BP.^7^,^28^ Today, the masters of treatment are the TKIs that are described and discussed, in details, in this issue of the journal.

## The Surrogate Markers of Outcome

The evaluation of the long term outcome (overall survival, OS, and progression-free survival, PFS) takes many years and does not help to adapt and to optimize the treatment in each single patient. Therefore, it is necessary to assess the hematologic, cytogenetic, and molecular responses as an early surrogate marker of the long term outcome (Table 1 and 2).^6^,^21^ The first step is the hematologic response (HR) that must be complete within the first 3 months. Although the utility of evaluating HR is obscured by the large use of HU before TKIs, any patient who for any reason is not yet in CHR at 3 months, must be considered as a failure, mandating a change of treatment.^6^ The second step is the cytogenetic response (CyR), and the third step is the molecular response (MR).^1^,^5^ The progress in technology and standardization of the MR have now made it possible to use only the MR.^6^,^29^–^32^ The correspondence between the CyR and the MR is not absolute but is fairly good. Whenever is possible, both responses should be considered, for the best assessment of the response.^6^ According to ELN recommendations, the “failure”, mandating a change of treatment, is defined: at 3 months as the lack of any CyR (Ph+>95%) and/or as a BCR-ABL1 transcripts level of more than 10%; at 6 months as the absence of a Partial CyR (PCyR, Ph+>35%) and/or as a BCR-ABL1 transcripts level > 10%; at 12 months as the absence of a complete CyR (CCyR, Ph+=0) and/or as a BCR-ABL1 transcripts level of more than 1% (Table 2).^6^ In all these cases, a change of treatment is mandatory. However, there are cases when the response is neither a failure nor optimal and is defined as “warning”, meaning that, in these cases, monitoring must be more frequent (Table 2).^6^ The optimal response to any first-line TKI treatment is shown in Table 3. In these cases, there are no indications for a change of treatment.^6^

## Standard Treatment, Chronic Phase, Firstline

Three TKIs are currently registered for the first-line treatment of chronic phase (CML), namely imatinib (Gleevec or Glivec, Novartis Pharma), nilotinib (Tasigna, Novartis Pharma), and dasatinib (Sprycel, Brystol-Myers Squibb). The recommended doses are 400 mg once daily, 300 mg twice daily, and 100 mg once daily, respectively. A higher dose of imatinib (300 to 400 mg twice daily), a combination of imatinib 400 mg once daily with rIFNα, and a higher dose of nilotinib (400 mg twice daily), have been reported to be also very effective, but cannot be considered as standard treatment.^11^–^13^,^22^,^23^,^33^–^36^ Two prospective, company-sponsored studies comparing imatinib with nilotinib^11^–^13^ and with dasatinib^14^,^15^ have reported a superiority of the two second generation TKIs, particularly in terms of MR rate, speed, and depth, with marginal benefit in PFS. These data are sufficient to include nilotinib and dasatinib in standard treatment recommendations, but are not enough to remove imatinib.^6^,^21^,^37^ There are not efficacy data that can help to make a decision between nilotinib and dasatinib. Therefore, the choice of standard treatment is based on the availability and the cost of the three drugs, on the professional experience of the prescriber, and on patient health status and comorbidities. A history, or a condition of high risk of arterial disease, diabetes, and pancreatic disease, can make the prescription of nilotinib problematic.^6^,^38^–^42^ A history, or a condition of high risk of hemorrhage, of respiratory diseases, autoimmune diseases, and infections complications, can make the prescription of dasatinib problematic.^6^,^39^,^40^,^43^–^45^ A history, or a condition of high risk of cardiac diseases, must be a warning for all three TKIs,^46^,^47^ and requests to initiate the treatment at a dose lower than that recommended.

## Standard Treatment, Chronic Phase, Second-Line

In the case of intolerance, it is recommended to switch to anyone of the other TKIs approved for first-line. The choice will depend on the side-effects of the first TKI, and on patient health status.

In the case of primary or secondary failure, if the first-line drug were imatinib, the choice will be between nilotinib and dasatinib; if the first-line drug were nilotinib or dasatinib, the other second generation TKI not already used, plus bosutinib and ponatinib should be considered. The change must be preceded by a mutational analysis, because the identification of a BCR-ABL1 mutation helps in the choice of the new treatment.^6^,^48^,^49^ The ten most frequent mutations, and their sensitivity to the TKIs are listed in Table 5. If the mutation is T315I, the choice will be always ponatinib, even if the first-line was imatinib.^6^,^17^,^50^,^51^

In the case of failure or intolerance to two TKIs, the choice will be anyone of the remaining TKIs. In such a situation, ponatinib is an important option.^6^,^17^,^50^,^51^

## Standard Treatment, Accelerated or Blastic Phase Firstline

If the disease initiates in AP or BP, and the patient is TKI-naïve, the standard treatment is imatinib (300 or 400 mg twice daily) or dasatinib (140 mg once daily or 70 mg twice daily) or nilotinib (400 mg twice daily). The response is assessed as for CP (Tables 1, 2 and 3).

If the disease progresses to AP or BP during TKI treatment, the choice is one of the TKIs that was not used in CP, with a preference for ponatinib. The response is always assessed as for CP (Tables 1, 2, and 3).

## Allogeneic Stem Cell Transplantation

AlloSCT is recommended:^6^

- For all the patients in BP at diagnosis or who progress to BP after TKI treatment, provided that a remission has been induced. AlloSTC in full BP is usually ineffective.
- For all the patients in AP at diagnosis not achieving an optimal response to the first line TKI or who progress to AP after TKI treatment (Table 3)
- For all CP patients who fail two TKIs and do not achieve an optimal response (Table 3) to the third line TKI
- For selected CP patients, who have high risk characteristics, fail the first TKI, and do not achieve optimal response (Table 3) to the second line TKI.

In all cases, a patient must be eligible for alloSCT. The definition of eligibility is never absolute because it is based on the balance between the risk of the disease and the risk of alloSCT.

## Experimental Treatment

There are three categories of patients eligible for an experimental treatment.

The first category is that of the patients who fail TKIs and alloSCT, or cannot be transplanted. These patients need effective treatment, but such a treatment has not yet been found.^27^

The second category is that of the patients who fall in the “warning” definition of the response. They can do well if they continue the treatment, but they can do as well or better if treatment is changed. Examples are the trial protocols testing an early switch from imatinib to second generation TKIs, when the BCR-ABL1 transcripts level at 3 months is more than 10%.^52^–^54^

The third category is that of the patients who achieve an optimal response to first line treatment, but never achieve an MR as deep as it is necessary to try to discontinue therapy and achieve a treatment-free remission.^55^ Examples are the trial protocols testing 2^nd^ generation TKIs frontline, or a late switch from imatinib to second generation TKIs.^56^,^57^

The fourth category is that of the patients who achieve a stable optimal response and are eligible for a trial of treatment discontinuation or reduction^58^,^59^

A separate issue is that of the clinical value of the combination of TKI with IFNα. That combination is currently tested frontline in several prospective studies, to evaluate if it may have a favorable effect on response rate, PFS, and treatment-free remission.^22^–^24^,^57^

## Discussion and Conclusions

Imatinib produced a big breakthrough in the course of CML. Today, after less than 15 years, we know more, we have more, and we want more. If, on one hand, we must realistically stay at standard treatment recommendations, on the other hand, we should continue to design new treatment protocols and to enroll new patients in prospective studies. This is not easy, because of the high efficacy of standard treatment. In any case, the treatment of CML must be guided by healthcare professionals with specific training and specific interest in CML, that are necessary for the optimization of the treatment and a proper utilization of the resources. The home physicians must be involved more and more in the care of the CML patient, because an optimal treatment ensures an average life expectancy, and the patient should no longer be considered as a patient at risk of dying of cancer, but as any other individual, at risk of developing complications and comorbidities of any type, that could also be triggered by the treatment itself. In some patients, particularly in the elderly where comorbidities are frequent and important and concomitant medications can create problems, treatment can be temporarily discontinued or reduced,^55^,^58^ and in some patients the next ambitious step is to move towards a treatment-free remission.^59^ Finally, the voice of the patient should deserve a major attention. The side effects of TKIs are reported as “tolerable” and “manageable”, but when the side-effects, even minimal, even mild, are chronic, the quality of life and the compliance to treatment will be affected.^19^,^20^,^60^–^64^ There are now new goals for the assessment of the quality of life and the symptoms burden, like the EORTC QLQ-CML24 and the MDASI-CML questionnaires.^19^,^65^–^68^ They should be regularly administered to allow the patients to contribute actively to treatment optimization.

## Figures and Tables

**Table 1:** Definition of response. The CCyR can be defined either by chromosome banding analysis (CBA) of at least 20 marrow cell metaphases, or by interphase fluorescence-in-situ-hybridization (I-FISH) of at least 200 marrow or blood nuclei (6). The other CyRs can be defined only by CBA of marrow cell metaphases. Molecular response must be assessed by standardized RT-Q-PCR of RNA extracted from buffy coat blood cells and must be expressed according to the International Scale (IS) (6,28–31).

Hematologic Response (HR):	Complete: WBC < 10×10^9^/L, Platelet count < 450×10^9^/L, no immature granulocytes in the differential, and spleen non palpable.
Cytogenetic Response (CyR):	Complete (CCyR): no Ph+ metaphases or less than 1% BCR-ABL1 positive nuclei by I-FISH Partial (PCyR): Ph+ metaphases 1–35% Minor/Minimal (mCyR): Ph+ metaphases 36–95% None: Ph+ metaphases > 95%
Molecular response (MR):	MR^4.5^: BCR-ABL1 ≤ 0.003% (IS) MR^4.0^: BCR-ABL 1≤ 0.01% (IS) MR^3.0^ (Major MR, MMR): BCR-ABL1 < 0.1% (IS)

**Table 2:** Definition of response (failure or warning) to first line TKI treatments, according to ELN 2013 recommendations (6). “Failure” mandates for a change of treatment. “Warning” warns that the response must be monitored more frequently.

		HEMATOLOGIC RESPONSE	CYTOGENETIC RESPONSE	MOLECULAR RESPONSE
FAILURE	3 months	Not full	None (Ph+ > 95%)	
	6 months		Less than partial (Ph+>35%)	BCR-ABL >10%
	12 months		Less than complete (Ph+>0)	BCR-ABL >1%

WARNING	3 months		Minor/minimal (Ph+ 36–95%)	BCR-ABL >10%
	6 months		Partial (Ph+1–35%)	BCR-ABL 1–10%
	12 months			BCR-ABL 0.1–1%

**Table 3:** Definition of an optimal response, according to ELN 2013 recommendations.^6^ Optimal response means that the treatment, whatever it is, must be continued.

**3 months**	BCR-ABL1 ≤ 10% (IS) and/or at least PCyR (Ph+ ≤ 35%)
**6 months**	BCR-ABL1 < 1% (IS) and/or CCyR (Ph+ 0)
**12 months**	MR^3.0^ or MMR (BCR-ABL1 ≤ 0.1% (IS)
